# Draft genome sequence of epiphyte *Paenibacillus kyungheensis* strain Cap286 isolated from *Chenopodium album* L. leaves

**DOI:** 10.1128/mra.00383-26

**Published:** 2026-05-06

**Authors:** Vladimir K. Chebotar, Maksim R. Losev, Maria S. Gancheva, Dmitriy V. Kudryavtcev, Olga A. Bortsova, Elena P. Chizhevskaya, Veronika N. Pishchik

**Affiliations:** 1 All-Russia Research Institute for Agricultural Microbiology 117473, St. Petersburg, Pushkin, Russia; 2 Saint Peterburg State University, St. Petersburg, Russia; Rochester Institute of Technology, Rochester, New York, USA

**Keywords:** genome sequence, epiphyte, *Paenibacillus kyungheensis*, *Chenopodium album*, leaves

## Abstract

Here, we report the genome sequence of the epiphytic *Paenibacillus kyungheensis* bacterium isolated from *Chenopodium album* L. leaves. The raw sequencing data were obtained using the NextSeq 1000 technology (Illumina). The assembly comprises two scaffolds, totaling 5,257,300 bp with a guanine-cytosine content of 39%.

## ANNOUNCEMENT

*Paenibacillus kyungheensis* strain Cap286, an epiphytic gram-positive bacterium, was isolated from the surface of the leaves of *Chenopodium album* collected in the Stavropol region of Russia (N_45°23′36″, E_41°45′52″) by placing plant leaf material (10 g) in 100 mL of sterile water and subjecting it to ultrasonic treatment (Bandelin; 50 Hz) for 10 min. Aliquots (0.1 mL) of serial dilutions were plated onto LB agar (Sigma-Aldrich, USA) and incubated for 3–5 days at 28°C.

Genomic DNA was extracted from single colony using the Wizard Genomic DNA Purification Kit (Promega, USA). A sequencing library was then prepared with the KAPA HyperPlus Kit (Roche, Switzerland). Purification was performed using KAPA Hyperpure beads (Roche, Switzerland). The quality and size distribution of the library were assessed with the High-Sensitivity DNA Kit (Agilent Technologies, USA), while its concentration was determined using the Quant-iT DNA Assay Kit (Thermo Fisher Scientific, USA). The sample was sequenced on an Illumina NextSeq 1000 instrument using a NextSeq 1000/2000 P1 Reagent Kit (300 cycles), which produced 2,075,020 paired-end reads with an average length of 150 bp.

The quality of the reads was checked using FastQC v0.11.9 (1). The draft genome was obtained with the SPAdes v4.2.0 genome assembler (2). FastANI v1.34 (3) identified that the closest reference genome was the genome of *Paenibacillus kyungheensis* GCF_028606985.1 (97.5% average nucleotide identity [ANI]) from the National Center for Biotechnology Information (NCBI) database (4). Scaffolding was performed using Ragtag v2.1.0 (5). Contigs shorter than 1 kbp were removed using the reformat tool from BBMap v39.08 (https://sourceforge.net/projects/bbmap/). Reads were mapped to the assembly using bwa v0.7.18 (6), and read coverage was calculated using the samtools coverage tool v1.13 (7). Assembly statistics were evaluated using QUAST v5.1.0 (8). The final assembly consisted of two scaffolds with a total length of 5,257,300 bp, guanine-cytosine content of 38.93%, N50 of 5,256,028, and average genome coverage of 64.8×. The completeness of genome was evaluated using BUSCO v5.2.2 (9) with the bacillales_odb10 lineage data set, and the BUSCO score was 99.8% (complete BUSCOs). At the same time, CheckM v1.2.3 (10) indicated 87.73% completeness and 1.7% contamination. A total of 4,683 genes were predicted on the assembly by NCBI Prokaryotic Genome Annotation Pipeline (PGAP) v6.10 (11). Unless otherwise noted, default parameters were used for all software.

Secondary metabolite biosynthetic gene clusters (BGCs) were identified using antiSMASH v8.0 (12). The analysis revealed eight predicted clusters, including several for ribosomally synthesized and post-translationally modified peptides (RiPPs). Among these, a cluster for paeninodin was predicted. Paeninodin is a member of the lasso peptide class known for diverse bioactivities, such as antimicrobial, antiviral, and enzyme inhibitory functions (13). A separate cluster was predicted for the biosynthesis of a Class II lanthipeptide, mersacidin, which inhibits peptidoglycan synthesis (14). Additional clusters were associated with carotenoid and siderophore biosynthesis.

The significant role of Cap286 in improving wheat drought tolerance (15) underscores the importance of the genomic data presented here. This genome assembly provides a critical resource for future studies aimed at understanding, optimizing, and potentially engineering this strain for sustainable agriculture.

## Data Availability

The genome sequence and raw reads were deposited in NCBI and can be accessed under BioProject PRJNA1337296 (SRA SRR35708438, assembly GCF_053570845.1).
